# Novel Mode of Molybdate Inhibition of *Desulfovibrio vulgaris* Hildenborough

**DOI:** 10.3389/fmicb.2020.610455

**Published:** 2020-12-08

**Authors:** Grant M. Zane, Judy D. Wall, Kara B. De León

**Affiliations:** Division of Biochemistry, University of Missouri, Columbia, MO, United States

**Keywords:** sulfate‐reducing bacteria, molybdate inhibition, oxyanion, ATP depletion, Desulfovibrio, YcaO

## Abstract

Sulfate-reducing microorganisms (SRM) are found in multiple environments and play a major role in global carbon and sulfur cycling. Because of their growth capabilities and association with metal corrosion, controlling the growth of SRM has become of increased interest. One such mechanism of control has been the use of molybdate (MoO_4_^2−^), which is thought to be a specific inhibitor of SRM. The way in which molybdate inhibits the growth of SRM has been enigmatic. It has been reported that molybdate is involved in a futile energy cycle with the sulfate-activating enzyme, sulfate adenylyl transferase (Sat), which results in loss of cellular ATP. However, we show here that a deletion of this enzyme in the model SRM, *Desulfovibrio vulgaris* Hildenborough, remained sensitive to molybdate. We performed several subcultures of the ∆*sat* strain in the presence of increasing concentrations of molybdate and obtained a culture with increased resistance to the inhibitor (up to 3 mM). The culture was re-sequenced and three single nucleotide polymorphisms (SNPs) were identified that were not present in the parental strain. Two of the SNPs seemed unlikely candidates for molybdate resistance due to a lack of conservation of the mutated residues in homologous genes of closely related strains. The remaining SNP was located in DVU2210, a protein containing two domains: a YcaO-like domain and a tetratricopeptide-repeat domain. The SNP resulted in a change of a serine residue to arginine in the ATP-hydrolyzing motif of the YcaO-like domain. Deletion mutants of each of the three genes apparently enriched with SNPs in the presence of inhibitory molybdate and combinations of these genes were generated in the Δ*sat* and wild-type strains. Strains lacking both *sat* and DVU2210 became more resistant to molybdate. Deletions of the other two genes in which SNPs were observed did not result in increased resistance to molybdate. YcaO-like proteins are distributed across the bacterial and archaeal domains, though the function of these proteins is largely unknown. The role of this protein in *D. vulgaris* is unknown. Due to the distribution of YcaO-like proteins in prokaryotes, the veracity of molybdate as a specific SRM inhibitor should be reconsidered.

## Introduction

The sulfate-reducing microorganisms (SRM) are a diverse group of bacteria and archaea capable of performing dissimilatory reduction of sulfate to sulfide. SRM reduce sulfate by a dissimilatory reduction process that results in a membrane proton gradient that supports ATP synthesis for the bacterium. The enzymes required for this metabolism include transport of sulfate (the proteins which are as yet unidentified), activation of sulfate to adenosine 5'-phosphosulfate (APS) by sulfate adenylyl transferase (Sat), reduction of APS to bisulfite by APS reductase (ApsBA), and reduction of bisulfite to sulfide by dissimilatory bisulfite reductase (DsrAB; Postgate, 1984). Additionally, there are transmembrane protein complexes that interact specifically with the above enzymes to provide electrons for reduction (i.e., QmoABC is the sole provider of electrons to ApsBA and DsrMKJOP is thought to be the provider of electrons to DsrAB; Venceslau et al., 2010; Zane et al., 2010).

The SRM are found in many environments and are key players in the corrosion of metal and concrete. This corrosion often results in a loss of structural integrity of these surfaces leading to high costs of replacement, repair, and maintenance. Because of these detrimental effects, inhibitors of SRM are often employed in industrial systems. Analogs of sulfate are competitive inhibitors of sulfate reduction and include molybdate (Taylor and Oremland, 1979), tungstate (Taylor and Oremland, 1979), selenate (Postgate, 1949), monofluorophosphate (Postgate, 1952; Carlson et al., 2015), and others. These inhibitors are reported to be specific to SRM (reviewed in Oremland and Capone, 1988; Tanaka and Lee, 1997).

Molybdate (MoO_4_^2−^) has been used frequently as a specific inhibitor of SRM in environmental studies (Oremland and Taylor, 1978; Nedwell and Azni bin Abdul Aziz, 1980; Balba and Nedwell, 1982; Carlson et al., 2015). Molybdate inhibition is thought to be due to futile cycling that depletes intracellular ATP (Taylor and Oremland, 1979). In *Desulfovibrio vulgaris* ATCC 7757, ATP levels were 45% of the control when 10 mM molybdate was present (Taylor and Oremland, 1979). The first enzyme in sulfate reduction, Sat, activates molybdate forming an unstable analog to APS, adenosine 5'-molybdophosphate, which decomposes in water to molybdate and AMP (Wilson and Bandurski, 1958; Oremland and Capone, 1988). Molybdate may also inhibit sulfate transport in SRM, though reports are conflicting (Newport and Nedwell, 1988; Cypionka, 1989). Therefore, the exact mode of inhibition has not been shown to be unequivocally specific to SRM. The evidence that molybdate is a specific inhibitor of SRM remains incomplete, since not all organisms have been (nor can be) tested. In order to show that an inhibitor acts specifically against SRM, the target must be identified and shown to be specific to SRM. Alternatively, to show that one of these compounds is not a specific inhibitor of SRM, inhibition of one or more non-SRMs by molybdate should be shown.

If the mode of action for molybdate on SRM is solely due to futile cycling with Sat, it stands to reason that a mutant lacking Sat should be resistant to molybdate. Here, we show that a Δ*sat* mutant of the model SRM, *D. vulgaris* Hildenborough was still sensitive to molybdate. We then adapted a culture of the Δ*sat* mutant to be resistant to 3 mM sodium molybdate. Sequence variants within the culture identified three other proteins potentially involved in molybdate sensitivity. A deletion mutant was constructed for each combination of single, double, triple, and quadruple mutants of the genes encoding these three proteins and Sat to test the phenotype on molybdate. Only those strains lacking both *sat* and the gene at locus DVU2210 encoding a YcaO-like tetratricopeptide repeat protein showed resistance to molybdate. Deletions of either *sat* or DVU2210 alone showed inhibition of growth in the presence of molybdate. As homologs of DVU2210 are found throughout bacteria, this puts into question the assumption that molybdate inhibition is unique to SRM because of the sulfate activation activity.

## Materials and Methods

### Strains, Media, and Growth Conditions

The strains and plasmids used in this study are listed in Supplementary Table 1 and are available upon request. *Escherichia coli* strains were grown in 50 ml flasks containing 5 ml LC medium (Zane et al., 2010), incubated at 37°C, and shaken at 200 rpm. Agar (1.5% w/v) was added to LC for solidified medium. Where indicated, kanamycin or spectinomycin was added to LC medium to a final concentration of 50 or 100 μg/ml, respectively (Gold Biotechnology, Inc., St. Louis, MO).

*Desulfovibrio vulgaris* strains were grown in an anoxic atmosphere at 34°C in MO medium modified from Zane et al. (2010) and containing the following: 8 mM magnesium chloride, 20 mM ammonium chloride, 0.6 mM calcium chloride, 2 mM phosphate buffer (pH 7.2), 30 mM Tris-HCl (pH 7.4), 6 ml/L trace elements, 1 ml/L vitamin solution, and 0.48 ml/L iron solution. The trace element’s stock solution contained 2.5 mM manganese chloride, 1.26 mM cobalt chloride, 1.47 mM zinc chloride, 210 μM sodium molybdate, 320 μM boric acid, 420 μM nickel chloride, 11.7 μM cupric chloride, 23 μM sodium selenite, and 24 μM sodium tungstate. The vitamin solution was made at 10x the concentrations described in Brandis and Thauer (1981). The iron solution was made by dissolving 125 mM ferrous chloride in a 250 mM solution of EDTA. When indicated, media contained yeast extract [designated as Y, 0.1% (w/v)], lactate (L, 60 mM), pyruvate (P, 60 mM), sulfate (S4, 30 mM), and/or sulfite (S3, 20 mM). When grown fermentatively, cysteine hydrochloride (c, 0.5 mM) was added as a sulfur source. Thus, as an example of the medium designations, MO medium containing yeast extract, pyruvate, and cysteine was called MOYPc and the addition of 30 mM sulfate to this medium was called MOYPcS4. All MO media contained sodium thioglycolate (1.2 mM) as a reductant and was adjusted to pH 7.2 with 5 M hydrochloric acid. To grow colonies in solidified medium, 1.5% (w/v) agar was added before autoclaving. Sulfite stock solutions were sterilized by filtration and added after the medium had been autoclaved. Sterile sodium molybdate, sodium tungstate, G418 (400 μg/ml, Gold Biotechnology, Inc.), spectinomycin (100 μg/ml), or 5-fluorouracil [5FU, 40 μg/ml, (Acros Organics, Geel, Belgium)] was added to the medium after autoclaving where indicated. Freezer stocks of bacterial cultures were prepared by adding glycerol to a final concentration of 10% (v/v) and stored at −80°C.

To assess growth of *D. vulgaris* under different medium conditions, 5 ml of prepared medium was dispensed into 18 × 150 mm Balch tubes (Chemglass Life Sciences, Vineland, NJ) which were then flushed with nitrogen gas, closed with a rubber stopper, and sealed with an aluminum cap. Tubes containing medium were autoclaved. Freezer stocks of *D. vulgaris* strains were thawed, and 0.5 ml used to inoculate 5 ml of MOYPc anoxic medium amended with 2 mM sulfite. These cultures were allowed to grow for 2 days and then used to inoculate experimental cultures to track growth by optical density at 600 nm. The volume of cells used to inoculate each tube was adjusted depending on the optical density of the starter culture so that approximately 5 × 10^7^ cells were inoculated into each tube.

To adapt a culture of the *sat* deletion strain, JW9271, to molybdate, a 1 ml freezer stock of this strain was grown in 10 ml MOYPc medium with 0.1 mM molybdate (defined as passage 0). When growth was observed, the culture was subcultured (0.5 ml into 5 ml MOYPc with 0.1 mM molybdate). Upon the fifth passage, an additional culture was inoculated into MOYPc containing 1 mM molybdate. Upon the twelfth passage, the culture maintained in 1 mM molybdate was subcultured into media containing 1 mM or 3 mM molybdate. The culture was stably resistant to 3 mM molybdate from the twelfth passage forward. DNA from the culture grown at the twelfth passage was sequenced (see below).

### Plasmid and Strain Construction

Plasmids (Supplementary Table 1) were constructed by sequence and ligation independent cloning, SLIC (Li and Elledge, 2007), with methods described previously (De León et al., 2017). In short, the plasmids were designed to contain the pUC origin of replication, the spectinomycin-resistance gene (*aadAI*), a PCR amplicon of the region upstream of the gene(s) to be deleted (upstream), a PCR amplicon of the region downstream of the gene(s) to be deleted (downstream). In the marker-exchange plasmids, a two-gene synthetic operon (*P_npt_-npt-upp*) containing a kanamycin-resistance gene, neomycin phosphotransferase II (*npt*) expressed under its native promotor (P_npt_), and uracil phosphoribosyltransferase (*upp*) was placed between the upstream and downstream fragments on the plasmid. In plasmids for markerless deletion, the upstream and downstream regions of the gene were immediately adjacent to each other so that an in-frame, markerless deletion would be generated from recombination with this plasmid in the marker exchange mutant. PCR amplification of the fragments for SLIC was performed with the DNA polymerase Herculase II (Agilent Technologies, Santa Clara, CA). The primers used to construct the plasmids for this study were purchased from Integrated DNA Technologies, Inc. (Coralville, IA) and are listed in Supplementary Table 2. After the SLIC reaction, equimolar concentrations of the cleaned PCR products were transformed into silver efficiency, α-select *E. coli* cells as per manufacturers’ guidelines (Bioline, London, United Kingdom). Transformants were selected on LC plates containing either kanamycin (for marker-exchange plasmids) or spectinomycin (for marker-less deletion plasmids). Colonies were screened by PCR with Taq polymerase [New England Biolabs, Inc. (NEB), Ipswich, MA]. The upstream and downstream regions were sequenced at the DNA Core facilities at the University of Missouri and compared with the published sequence to avoid introduction of unintended PCR errors.

The marker-exchange plasmids were electroporated into the Δ*upp* parental strain JW710 or a derivative with a markerless deletion of one or more additional gene(s) as described previously (Zane et al., 2010) with the modifications below. An overnight culture of the parental strain was prepared by inoculating 35 ml of medium with a 1 ml freezer stock and incubating at 34°C. If the strain to be transformed was lacking *sat*, MOYLS3 was used in the place of MOYLS4. Transformed cells were allowed to recover overnight at 34°C in 1 ml MOYLS4 or MOYLS3. Three different volumes (10, 100, and 900 μl) of the recovered cells were each placed in an empty Petri dish. Cooled, molten MOYLS4 or MOYLS3 agar was poured into the Petri dish, and the suspension was mixed by swirling the Petri dish in a “figure-8 pattern” before the medium solidified. Plates were incubated in an anoxic box containing anaero-packs (Mitsubishi Gas, Chemical Co., Inc., Tokyo, Japan) at 34°C for 4 days. Antibiotics were included for the selection of the marker-exchange deletion strain (G418) or the marker-less deletion strain (5FU). Putative transformants for marker exchange deletions were screened by patching isolates onto MOYLS4 or MOYLS3 agar plates containing either spectinomycin, G418, 5FU, or no antibiotic. This allowed identification of isolates that contained an integrated plasmid (Sp^r^, G418^r^, and 5FU^s^) or were contaminants from the parental strain (5FU^r^ but no additional antibiotic resistances). The process was repeated with the marker-exchange deletion as a recipient for transformation of a markerless deletion plasmid to create the targeted markerless deletion mutant.

All strains (Supplementary Table 1) were verified by Southern blotting as described previously (Zane et al., 2010) with restriction enzymes from NEB. For the deletion of DVU2210, the enzyme MspA1I was used to distinguish between band sizes of 1,904 bp (wild-type), 1,152 bp (marker-exchange), and 1,453 bp (marker-less deletion). For the deletion of DVU1975, two different Southern blot confirmations were performed. One of the Southern blots was with the restriction enzyme EcoRV and the other with HincII to distinguish between band sizes of 1,664 bp and 2,294 bp (wild-type), 3,947 bp and 1,211 bp (marker-exchange), and 2,328 bp and 1,559 bp (marker-less deletion), respectively. For the deletion of DVU2305-6, the enzyme SalI was used to distinguish between band sizes of 5,371 bp (wild-type), 2,590 bp (marker-exchange), and 3,738 bp (marker-less deletion).

### Protein Yield Determination

Protein was determined with the Bradford assay (Bradford, 1976), and bovine serum albumin (Sigma, St. Louis, MO) was used to prepare solutions of protein standards.

### Sequencing of Molybdate-Resistant Culture

Sequencing of the molybdate-resistant culture was performed at the University of Missouri DNA Core facilities. Genomic DNA was isolated and prepared as described previously (De León et al., 2017). Raw sequences were mapped to the *D. vulgaris* Hildenborough genome (NCBI reference accession no. NC_002937.3 and NC_005863.1) with Bowtie 2 (Langmead and Salzberg, 2012) within Geneious (v.8.1.8; Biomatters, Ltd., Auckland, New Zealand). Sequence variants occurring at ≥25% frequency were identified within Geneious. The dataset for this study can be found in the NBCI Sequence Read Archive (SRA) database with the BioProject Accession Number PRJNA665718.

## Results

### Deletion of *sat* Does Not Protect *Desulfovibrio vulgaris* From Molybdate Inhibition

**Figure 1:**
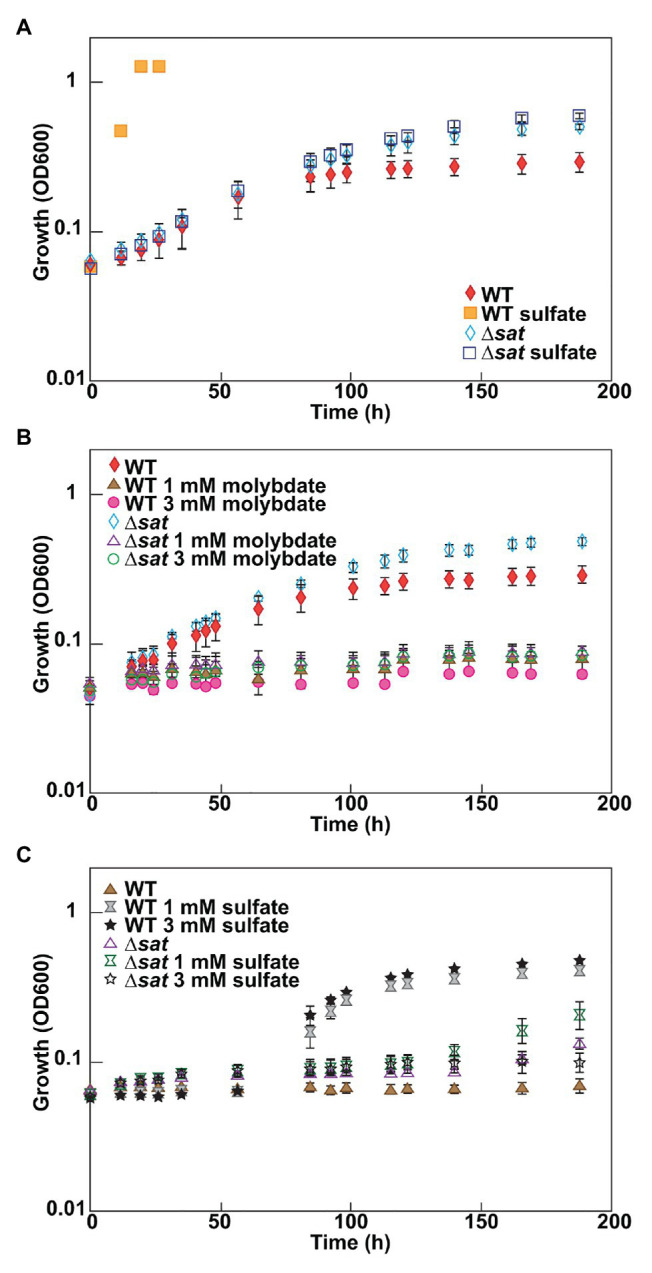
*Desulfovibrio vulgaris* wild type and Δ*sat* strains grown in MOYPc with different amendments. Cultures were amended with **(A)** 0 or 30 mM sulfate, **(B)** 0, 1, or 3 mM molybdate, and **(C)** 1 mM molybdate plus 0, 1, or 3 mM sulfate. Growth was measured by optical density at 600 nm (OD600). Error bars denote the standard deviation across triplicates.

As molybdate is considered a specific inhibitor of the sulfate-reducing bacteria by depletion of intracellular ATP from a futile cycle with the sulfate-activating enzyme, Sat (Taylor and Oremland, 1979), it was expected that a deletion of *sat* should result in a molybdate-resistant strain. A ∆*sat* strain of *D. vulgaris* had previously been constructed (Hillesland et al., 2014) and was unable to respire sulfate. The resistance of this strain to molybdate was tested under fermentative conditions, and compared to that of JW710, the parental strain used as wild type (WT) in this study. Both of these strains grew at similar rates in fermentative conditions, though the ∆*sat* strain reached a higher maximum optical density (Figure 1A). Thus, it appears that the presence of Sat, when sulfate is not present, does cost the cell some growth power. The presence of sulfate did not have a growth effect in the ∆*sat* strain. Yet, surprisingly, the ∆*sat* strain remained sensitive to molybdate (Figure 1B). In contrast, the ∆*sat* strain was moderately resistant to 5 mM tungstate, another sulfate analog (Supplementary Figure 1).

### Protective Nature of Sulfate to Molybdate Stress in *Desulfovibrio vulgaris*

Since molybdate and sulfate both exhibit tetrahedral geometry and are often recognized by the same family of transporters (Aguilar-Barajas et al., 2011), it was hypothesized that high sulfate concentrations could have a protective effect against molybdate stress in the model sulfate-reducing bacterium *D. vulgaris*. To test this hypothesis, *D. vulgaris* strain JW710 was grown in MOYPc medium with 1 mM molybdate and increasing concentrations of sulfate (0, 1, and 3 mM; Figure 1C). The presence of sulfate did not provide a protective effect for *D. vulgaris* against molybdate initially, even with sulfate concentrations as high as 3 mM. However, there was a recovery in growth after about 40–50 h regardless of sulfate concentration. This delay could not be alleviated by supplementing the culture with 50 mM sulfate at zero time (data not shown). Yet, cultures without molybdate did not lag (see WT growth on MOYPS4c in Figure 1A). The growth inhibition of ∆*sat* strain by 1 mM molybdate was not alleviated by supplementing the culture with sulfate either (Figure 1C). This was interpreted to mean that added sulfate was not able to outcompete the molybdate to relieve inhibition.

### Adaptation of the ∆*sat* Strain to Molybdate

**Figure 2:**
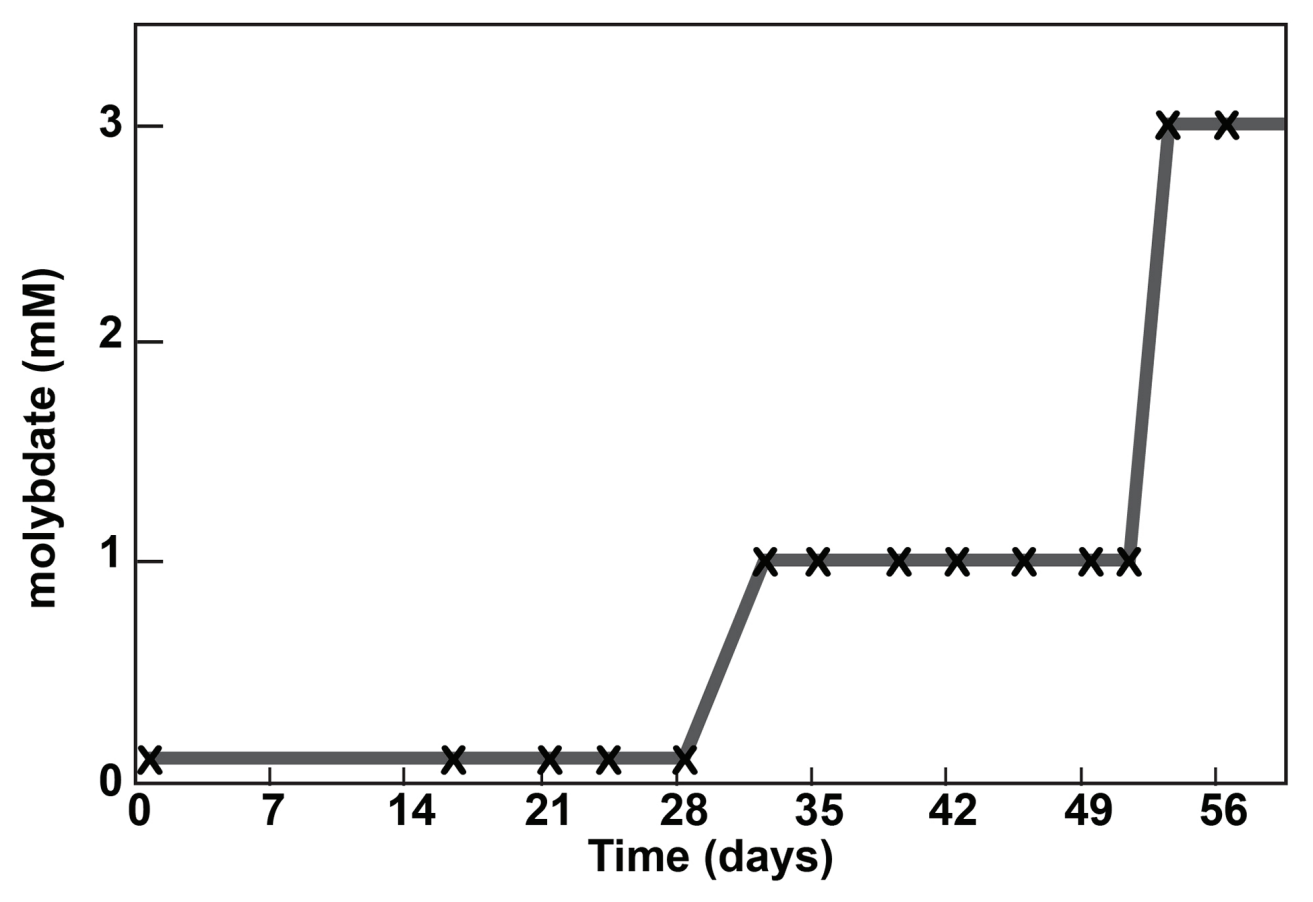
Adaptation of a Δ*sat* culture to molybdate. A culture was serially passaged in the presence of molybdate. Each subculture is marked with an “x”. The molybdate concentration into which the subculture was transferred is shown on the *y*-axis.

Due to the unexpected result that the Δ*sat* strain remained sensitive to molybdate, a culture of the strain was sequentially cultured in MOYPc medium with increasing amounts of molybdate (0.1, 1, and 3 mM; Figure 2). On Day 53, the culture was subcultured for the twelfth time, but this time it was transferred into 3 mM molybdate. The culture was resistant to 3 mM molybdate. The genome of this culture was sequenced at a depth of 65x average coverage across the chromosome. Three gene variants were found at 100% frequency in this population that were not present in the ancestral culture (Table 1). Each of the variants resulted in an amino acid change in a different encoded protein.

**Table 1:** Variants in the molybdate-adapted Δ*sat* culture.

Gene locus	Annotation	Nucleotide change	Amino acid change	Sequence coverage	Frequency of mutation in sequences (%)
DVU1975	methyl-accepting chemotaxis protein	G809A	A270V	63	100
DVU2210	YcaO-like, TPR domain protein	A583C	S195R	58	100
DVU2305	phosphate transport regulator	A491T	Q164L	52	100

The mutation in the chemotaxis protein (encoded at DVU1975) was not in a known conserved domain and was a conservative amino acid change (from alanine to valine). We considered this a low probability for resistance to molybdate. Secondly, a mutation in the phosphate transport regulator DVU2305 was found that might alter molybdate uptake. Finally, a SNP was found in DVU2210, which encodes a YcaO-like tetratricopeptide repeat (TPR) protein. This SNP was located in the region that aligns to the ATP-binding domain of the homologous *E. coli* YcaO (Ec_YcaO). The ATP-binding motif in YcaO proteins is Sx_6-7_Ex_3_Qx_3_ExxER (Dunbar et al., 2014). This motif is located at amino acids 184–203 in Ec_YcaO. In DVU2210, amino acids 221–236 are **S***AGNTEE***E***SIL***Q***GSC***E***LV***ER** (bold amino acids correspond to the conserved residues in the published YcaO ATP-binding domain, italicized amino acids correspond with the amino acids not conserved, and the location of the serine in DVU2210 mutated to arginine in the molybdate-resistant culture is underlined). This protein has not been extensively characterized. We hypothesized that this protein might be capable of adding adenosine phosphate to a molybdate molecule, thereby creating another futile cycle and depleting ATP in the cell. Other explanations are possible such as the YcaO domain activity was contributing to inhibition by molybdate because of the accumulation of a product and, when that protein was inactivated or deleted, at least partial resistance was established.

### Mutants Lacking *sat* and DVU2210 Are Resistant to Molybdate

**Figure 3:**
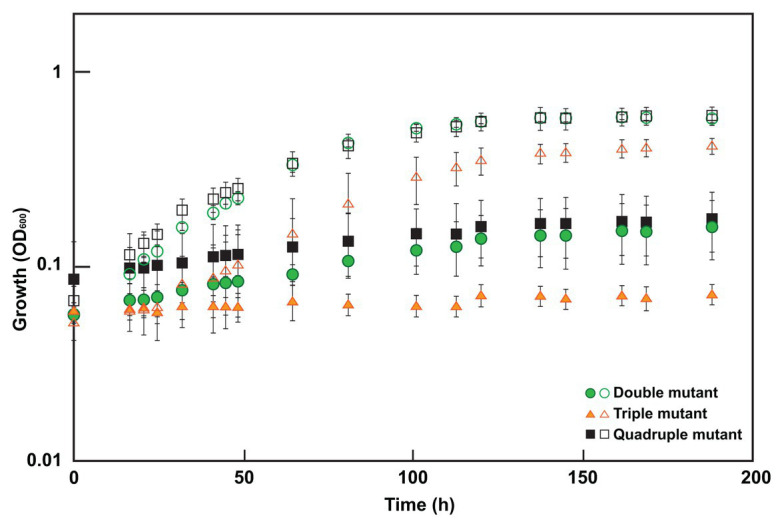
Growth of select mutants in 0 or 3 mM molybdate. Cultures were grown in MOYPc with (closed symbols) or without molybdate (open symbols). The double mutant lacked both *sat* and DVU2210, the triple mutant contained *sat* but lacked DVU2210, DVU1975, and DVU2305-6, and the quadruple mutant lacked all four of these genes. Growth was measured by optical density at 600 nm (OD600). Error bars denote the standard deviation across triplicates.

To determine which mutations were contributing to the molybdate resistant phenotype of the adapted Δ*sat* strain, markerless deletion mutants were constructed for all possible combinations of the four genes of interest (*sat* and the three genes with mutations in the genome resequencing; Supplementary Table 1). All mutants retained the ability to respire sulfite and those with *sat* still present in the genome also maintained the ability to respire sulfate. Each of these mutants was tested for resistance to 3 mM molybdate. Only those mutants lacking both *sat* and the gene DVU2210 encoding the YcaO-like TPR protein were resistant (Table 2; Figure 3). While the double mutant (Δ*sat* and ΔDVU2210) showed an increased resistance to 3 mM molybdate, it had a slower growth rate when compared to growth with 0 mM molybdate (Figure 3). The additional deletion of DVU1975 and/or DVU2305-6 did not increase the growth rate any further than the resistance observed in the double mutant lacking *sat* and DVU2210. Complementation of wild-type DVU2210 into the double mutant lacking both *sat* and DVU2210 restored the molybdate-sensitive phenotype (Table 2). By contrast, the strain complemented with a modified DVU2210 containing the mutation A583C conferring the amino acid change of S195R in the encoded protein was still resistant to 3 mM molybdate.

**Table 2:** Strain phenotype when grown fermentatively in MOYPc with 3 mM molybdate.

Category	Strain	Relevant genotype^a^				Phenotype with 3 mM molybdate^b^
		*sat* (DVU1295)	DVU1975	DVU2210	DVU2305-6	
Wild type	JW710	+	+	+	+	Sensitive
Single mutants	JW9271	−	+	+	+	Sensitive
	JW9505	+	−	+	+	Sensitive
	JW9479	+	+	−	+	Sensitive
	JW9253	+	+	+	−	Sensitive
Double mutants	JW9507	−	−	+	+	Sensitive
	JW9481	−	+	−	+	Resistant
	JW9513	−	+	+	−	Sensitive
	JW9517	+	−	−	+	Sensitive
	JW9521	+	−	+	−	Sensitive
	JW9519	+	+	−	−	Sensitive
Triple mutants	JW9511	−	−	−	+	Resistant
	JW9523	−	−	+	−	Sensitive
	JW9515	−	+	−	−	Resistant
	JW9525	+	−	−	−	Sensitive
Quadruple mutant	JW9527	−	−	−	−	Resistant
Complement and SNP strains	JW9498	+	+	+ complement	+	Sensitive
	JW9484	+	+	+ S195R mutation	+	Sensitive
	JW9499	−	+	+ complement	+	Sensitive
	JW9485	−	+	+ S195R mutation	+	Resistant

a + signifies gene is present in stain, − signifies gene is absent in strain.

b Resistance was defined as growth in MOYPc with 3 mM molybdate without an apparent lag as measured by optical density at 600 nm.

## Discussion

In this study, we show for the first time that a non-sulfate-reducing bacterium, a *D. vulgaris* strain lacking the sulfate activation protein Sat, is still sensitive to molybdate; thus, molybdate is not a specific inhibitor of sulfate reduction alone. By adapting this Δ*sat* strain to molybdate, a second target was identified that when activated by a SNP conferred molybdate sensitivity in combination with Δ*sat*. This was in DVU2210, a YcaO-like protein. YcaO proteins have been reported to use ATP to phosphorylate molecules, thereby facilitating cyclization, such as the phosphorylation of amide carbonyl oxygen during the cyclodehydration reaction in azoline formation (Dunbar et al., 2014). These proteins contain a unique ATP hydrolyzing domain. Interestingly, one of the mutations found in our molybdate-adapted Δ*sat* culture was in this domain. The deletion of DVU2210 or the mutated DVU2210 within the Δ*sat* background was sufficient to provide increased resistance to 3 mM molybdate (Figure 3; Table 2). We hypothesize that that the YcaO-like protein encoded by DVU2210 may activate molybdate and generate an unstable adenosine 5'-molybdophosphate, thereby creating a futile cycle that consumes ATP like that hypothesized for the sulfate-activating protein Sat. While the deletion of both *sat* and DVU2210 resulted in a strain resistant to 3 mM molybdate, there was still a growth effect when compared to the absence of molybdate. This suggests that there may be an additional target providing incremental molybdate inhibition and warrants further study. In contrast, the Δ*sat* strain was moderately resistant to 5 mM tungstate, another sulfate analog, suggesting that Sat is the main cause of tungstate sensitivity (Supplementary Figure 1). However, the quadruple mutant was resistant to tungstate, showing little to no difference in growth from the same strain grown without tungstate addition. Thus, one or more of these other genes (DVU2210, DVU1975, and DVU2305-6) may contribute to tungstate sensitivity.

The function of DVU2210 in *D. vulgaris* is unknown and the native target has not been determined. It is possible that, rather than acting directly on molybdate, another product of DVU2210 is causing molybdate sensitivity and, thus, the deletion provides some resistance. Mutations have been described to occur within DVU2210 in evolved cocultures of *D. vulgaris* Hildenborough and *Methanococcus maripaludis* in which interspecies electron transfer occurs in the absence of sulfate (Turkarslan et al., 2020). Interestingly, mutations in *sat* of *D. vulgaris* Hildenborough have also been described during evolution of these organisms in coculture and resulted in a loss of sulfate-reduction capacity of the *D. vulgaris* (Hillesland et al., 2014). To our knowledge, the coculture has not been tested for resistance to molybdate. Recent genome analysis of isolates from these evolved cocultures suggests that the mutations in *sat* and DVU2210 are mutually exclusive; both mutations are present in the community, but apparently not within the same cells (Turkarslan et al., 2020). Thus, we would predict that molybdate would still be inhibitory in the culture. However, it is possible that cells with mutations in both *sat* and DVU2210 are present but are rare in the population and, thus, not identified from end-point dilutions. In this case, molybdate addition would likely select for these populations.

The *D. vulgaris* and methanogen coculture evolution described previously (Turkarslan et al., 2020) and the Δ*sat* adaptation to molybdate in this study were both performed in the absence of sulfate and resulted in selection of mutations of DVU2210. We do not know if the absence of sulfate altered the selective pressure. It is possible that the selection for molybdate resistance might have had different results if adapted in the presence of sulfate even though the Δ*sat* strain cannot perform sulfate reduction. The ΔDVU2210 strain (containing *sat*) had a similar growth rate with sulfate respiration on MOYPS4 as the wild-type strain, so DVU2210 is not thought to play a direct role in sulfate reduction. It has been suggested that there may be a cost to the cells for expressing or having a functional copy of sulfate reduction genes when sulfate is not available (Hillesland et al., 2014). Our growth studies of the strains lacking either *sat* or DVU2210 support this; in both cases, the single mutants grew to a higher optical density during fermentation than the parental strain.

YcaO homologs are present throughout bacteria and archaeal lineages. There are over 9,000 YcaO members in InterPro (IPR003776) as of November 2020. It is not known how many, if any, of these homologs would cause sensitivity to molybdate; it is possible that only the *D. vulgaris* Hildenborough YcaO homolog or close relatives are sensitive. Nevertheless, because DVU2210 appears to augment sensitivity to molybdate, this does show that proteins not known to be involved in sulfate reduction may cause a bacterium to be sensitive to molybdate. In contrast, initial tests of YcaO-containing non-SRM strains do not support a clear role in sensitivity to molybdate. The anaerobic, non-SRM *Clostridium sporogenes* ATCC 7955 was resistant to 7.5 mM molybdate under the conditions tested even though it contains a YcaO-like protein encoded at gene locus LCABFAMN_01791 (Supplementary Figure 2). *E. coli* strains carrying a wild-type copy of DVU2210 on the plasmid pMO9482 or the modified DVU2210 on the plasmid pMO9483 were not sensitive to 10 mM molybdate in LC medium (Supplementary Figure 3A). These two examples might not query enough of the diversity of microorganisms and more research is necessary to assess molybdate sensitivity and the possible role of YcaO proteins in causing that sensitivity. Many bacteria, including *E. coli*, reduce sulfate by an assimilatory process, which uses many of the same enzymes as SRM (e.g., activation of sulfate to APS by Sat). Growth of *E. coli* and *Bacillus subtilis* has been shown previously to be inhibited by molybdate when sulfate is the source of sulfur (Pasternak, 1962). This was confirmed with our *E. coli* strains. When these strains were grown on defined medium M9 with 2 mM sulfate as the only available source of sulfur, 3 mM molybdate inhibited growth (Supplementary Figure 3B). The phenotype was similar with either *ycaO* gene, so the presence of wild-type DVU2210 was not interpreted to be the cause of molybdate sensitivity. Instead, the *E. coli* inhibition was likely due to futile cycling by Sat that depleted ATP. This showed that molybdate sensitivity could be the condition specific in organisms other than SRM.

That SRM are required to activate their substrate prior to use may make them more vulnerable to changes in ATP concentrations within the cell compared to other organisms. Sulfidogenesis has been shown to be 100-fold more sensitive to molybdate compared to general microbial growth in a marine enrichment (Carlson et al., 2015). The energy state of the environment may make the difference in whether molybdate specifically inhibits SRM or also inhibits others in the community. We have shown that Δ*sat* SRM growing by fermentation is sensitive to molybdate and mutations in both Sat and a second protein are needed to confer molybdate resistance. Homologs of the second protein, YcaO, are found broadly in prokaryotic lineages. Thus, the veracity of molybdate as a specific SRM inhibitor should be qualified. Additional microorganisms with YcaO protein domains should be tested for responses to molybdate. Future research will explore the question of possible suppressors of the deletion of *sat* from *D. vulgaris* selected in the presence of sulfate and the identification of the genes involved if suppression is found. That could be followed by an examination of molybdate inhibition for that putative function.

## Data Availability Statement

The datasets generated for this study can be found in NCBI BioProject accession PRJNA665718.

## Author Contributions

GZ, JW, and KD designed the study and developed methods. GZ and KD performed the research and wrote the manuscript. JW was responsible for funding acquisition. All authors analyzed data, contributed to manuscript revision, read, and approved the submitted version.

### Conflict of Interest

The authors declare that the research was conducted in the absence of any commercial or financial relationships that could be construed as a potential conflict of interest.

## References

[ref1] Aguilar-Barajas E. Díaz-Pérez C. Ramírez-Díaz M. I. Riveros-Rosas H. Cervantes C. (2011). Bacterial transport of sulfate, molybdate, and related oxyanions. Biometals 24, 687–707. 10.1007/s10534-011-9421-x, PMID: 21301930

[ref2] Balba M. T. Nedwell D. B. (1982). Microbial metabolism of acetate, propionate and butyrate in anoxic sediment from the Colne Point Saltmarsh, Essex, U. K. J. Gen. Microbiol. 128, 1415–1422. 10.1099/00221287-128-7-1415

[ref3] Bradford M. M. (1976). A rapid and sensitive method for the quantitation of microgram quantities of protein utilizing the principle of protein-dye binding. Anal. Biochem. 72, 248–254. 10.1016/0003-2697(76)90527-3, PMID: 942051

[ref4] Brandis A. Thauer R. K. (1981). Growth of *Desulfovibrio* species on hydrogen and sulphate as sole energy source. J. Gen. Microbiol. 126, 249–252.

[ref5] Carlson H. K. Stoeva M. K. Justice N. B. Sczesnak A. Mullan M. R. Mosqueda L. A.. (2015). Monofluorophosphate is a selective inhibitor of respiratory sulfate-reducing microorganisms. Environ. Sci. Technol. 49, 3727–3736. 10.1021/es505843z, PMID: 25698072

[ref6] Cypionka H. (1989). Characterization of sulfate transport in *Desulfovibrio desulfuricans*. Arch. Microbiol. 152, 237–243. 10.1007/BF00409657, PMID: 2476099

[ref7] De León K. B. Zane G. M. Trotter V. V. Krantz G. P. Arkin A. P. Butland G. P.. (2017). Unintended laboratory-driven evolution reveals genetic requirements for biofilm formation by *Desulfovibrio vulgaris* Hildenborough. mBio 8, e01696–e01617. 10.1128/mBio.01696-17, PMID: 29042504 PMC5646257

[ref8] Dunbar K. L. Chekan J. R. Cox C. L. Burkhart B. J. Nair S. K. Mitchell D. A. (2014). Discovery of a new ATP-binding motif involved in peptidic azoline biosynthesis. Nat. Chem. Biol. 10, 823–829. 10.1038/nchembio.1608, PMID: 25129028 PMC4167974

[ref9] Hillesland K. L. Lim S. Flowers J. J. Turkarslan S. Pinel N. Zane G. M.. (2014). Erosion of functional independence early in the evolution of a microbial mutualism. Proc. Natl. Acad. Sci. U. S. A. 111, 14822–14827. 10.1073/pnas.1407986111, PMID: 25267659 PMC4205623

[ref10] Langmead B. Salzberg S. L. (2012). Fast gapped-read alignment with Bowtie 2. Nat. Methods 9, 357–359. 10.1038/nmeth.1923, PMID: 22388286 PMC3322381

[ref11] Li M. Z. Elledge S. J. (2007). Harnessing homologous recombination *in vitro* to generate recombinant DNA via SLIC. Nat. Methods 4, 251–256. 10.1038/nmeth1010, PMID: 17293868

[ref12] Nedwell D. B. Azni bin Abdul Aziz S. (1980). Heterotrophic nitrogen fixation in an intertidal saltmarsh sediment. Estuar. Coast. Mar. Sci. 10, 699–702. 10.1016/S0302-3524(80)80097-1

[ref13] Newport P. J. Nedwell D. B. (1988). The mechanisms of inhibition of *Desulfovibrio* and *Desulfotomaculum* species by selenate and molybdate. J. Appl. Bacteriol. 65, 419–423. 10.1111/j.1365-2672.1988.tb01911.x

[ref14] Oremland R. S. Capone D. G. (1988). “Use of “specific” inhibitors in biogeochemistry and microbial ecology” in Advances in microbial ecology. ed. Marshall K. C. (New York: Springer, US), 285–383.

[ref15] Oremland R. S. Taylor B. F. (1978). Sulfate reduction and methanogenesis in marine sediments. Geochim. Cosmochim. Acta 42, 209–214. 10.1016/0016-7037(78)90133-3

[ref16] Pasternak C. A. (1962). Sulphate activation and its control in *Escherichia coli* and *Bacillus subtilis*. Biochem. J. 85, 44–49. 10.1042/bj0850044, PMID: 13941733 PMC1243909

[ref17] Postgate J. (1949). Competitive inhibition of sulphate reduction by selenate. Nature 164, 670–671. 10.1038/164670b0

[ref18] Postgate J. R. (1952). Competitive and non-competitive inhibitors of bacterial sulphate reduction. J. Gen. Microbiol. 6, 128–142. 10.1099/00221287-6-1-2-128, PMID: 14927859

[ref19] Postgate J. R. (1984). The sulphate-reducing bacteria. 2nd Edn. New York: Cambridge University Press.

[ref20] Tanaka S. Lee Y. -H. (1997). Control of sulfate reduction by molybdate in anaerobic digestion. Water Sci. Technol. 36, 143–150. 10.2166/wst.1997.0441

[ref21] Taylor B. F. Oremland R. S. (1979). Depletion of adenosine triphosphate in *Desulfovibrio* by oxyanions of group VI elements. Curr. Microbiol. 3, 101–103. 10.1007/BF02602440

[ref22] Turkarslan S. Stopnisek N. Thompson A. W. Arens C. E. Valenzuela J. J. Wilson J. (2020). Synergistic epistasis enhances cooperativity of mutualistic interspecies interactions. bioRxiv [Preprint]. 10.1101/2020.06.22.160184 PMC8319347 33612833

[ref23] Venceslau S. S. Lino R. R. Pereira I. A. C. (2010). The Qrc membrane complex, related to the alternative complex III, is a menaquinone reductase involved in sulfate respiration. J. Biol. Chem. 285, 22774–22783. 10.1074/jbc.M110.124305, PMID: 20498375 PMC2906268

[ref24] Wilson L. G. Bandurski R. S. (1958). Enzymatic reactions involving sulfate, sulfite, selenate, and molybdate. J. Biol. Chem. 233, 975–981. PMID: 13587526

[ref25] Zane G. M. Yen H. C. B. Wall J. D. (2010). Effect of the deletion of *qmoABC* and the promoter-distal gene encoding a hypothetical protein on sulfate reduction in *Desulfovibrio vulgaris* Hildenborough. Appl. Environ. Microbiol. 76, 5500–5509. 10.1128/AEM.00691-10, PMID: 20581180 PMC2918943

