# Supplementary material for: Novel Mode of Molybdate Inhibition of Desulfovibrio vulgaris Hildenborough
Source: Front Microbiol. 2020 Dec 8;11:610455. doi: 10.3389/fmicb.2020.610455 (PMC7774982; doi:10.3389/fmicb.2020.610455)
Supplement: Supplementary file 1 [file Data_Sheet_1.pdf]

Supplementary Material

Supplementary Table 1. Bacterial strains and plasmids used in this study

**Bacterial strain or plasmid**	**Relevant characteristic(s)[a] for these studies**	**Source or reference**
***Escherichia. coli* strains**		
α-select (silver efficiency)	*deoR endA1 recA1 relA1 gyrA96 hsdR17*(r_k^- m_k^+) *phoA supE44 thi-1* Δ(*lacZYA-argF*)*U169* φ80*lacZ*ΔM15 λ^- F^-	Bioline
Desulfovibrio vulgaris		
Hildenborough	Wild-type strain, ATCC29579, $5FU^s$ $G418^s$ Sp^s	ATCC 29579, received ca. 2003
JW710	*D. vulgaris* Hildenborough Δ*upp*; Km^s; $5FU^r$; parental strain	(Keller et al., 2009)
JW9251	JW710, ΔDVU2305-6::(P_{npt}-*npt-upp*); Km^r, $5FU^s$; marker exchange deletion	This study
JW9253	JW9251, ΔDVU2305-6 Δ(P_{npt}-*npt-upp*); Km^s; $5FU^r$; markerless deletion	This study
JW9271	JW710, Δ*sat*; markerless deletion	(Hillesland et al., 2014)
JW9477	JW710, ΔDVU2210::(P_{npt}-*npt-upp*); Km^r, $5FU^s$; marker exchange deletion	This study
JW9479	JW9477, ΔDVU2210 Δ(P_{npt}-*npt-upp*); Km^s; $5FU^r$; markerless deletion	This study
JW9480	JW9271, Δ*sat* ΔDVU2210::(P_{npt}-*npt-upp*); Km^r, $5FU^s$; marker exchange deletion	This study
JW9481	JW9480, Δ*sat* ΔDVU2210 Δ(P_{npt}-*npt-upp*); Km^s; $5FU^r$; markerless deletion	This study
JW9484	JW9477, (P_{npt}-*npt-upp*)::DVU2210$_{A583C}$; Km^s; $5FU^r$; creation of site-directed mutation in DVU2210	This study
JW9485	JW9480, Δ*sat* (P_{npt}-*npt-upp*)::DVU2210$_{A583C}$; Km^s; $5FU^r$; creation of site-directed mutation in DVU2210	This study
JW9498	JW9477, (P_{npt}-*npt-upp*)::DVU2210; complement, replacement of marker with wild-type DVU2210 gene Km^s; $5FU^r$	This study
JW9499	JW9480, Δ*sat,* (P_{npt}-*npt-upp*)::DVU2210; complement, replacement of marker with wild-type DVU2210 gene; Km^s; $5FU^r$	This study

JW9503	JW710, ΔDVU1975::(P_{npt}-*npt-upp*); Km^{r}, 5FU^{s}; marker exchange deletion	This study
JW9505	JW9503, ΔDVU1975 Δ(P_{npt}-*npt-upp*); Km^{s}; 5FU^{r}; markerless deletion	This study
JW9506	JW9271, Δ*sat* ΔDVU1975::(P_{npt}-*npt-upp*); Km^{r}, 5FU^{s}; marker exchange deletion	This study
JW9507	JW9506, Δ*sat* ΔDVU1975 Δ(P_{npt}-*npt-upp*); Km^{s}; 5FU^{r}; markerless deletion	This study
JW9510	JW9481, Δ*sat* ΔDVU2210 ΔDVU1975::(P_{npt}-*npt-upp*); Km^{r}, 5FU^{s}; marker exchange deletion	This study
JW9511	JW9510, Δ*sat* ΔDVU2210 ΔDVU1975 Δ(P_{npt}-*npt-upp*); Km^{s}; 5FU^{r}; markerless deletion	This study
JW9512	JW9271, Δ*sat* ΔDVU2305-6::(P_{npt}-*npt-upp*); Km^{r}, 5FU^{s}; marker exchange deletion	This study
JW9513	JW9512, Δ*sat* ΔDVU2305-6 Δ(P_{npt}-*npt-upp*); Km^{s}; 5FU^{r}; markerless deletion	This study
JW9514	JW9481, Δ*sat* ΔDVU2210 ΔDVU2305-6::(P_{npt}-*npt-upp*); Km^{r}, 5FU^{s}; marker exchange deletion	This study
JW9515	JW9514, Δ*sat* ΔDVU2210 ΔDVU2305-6 Δ(P_{npt}-*npt-upp*); Km^{s}; 5FU^{r}; markerless deletion	This study
JW9516	JW9479, ΔDVU2210 ΔDVU1975::(P_{npt}-*npt-upp*); Km^{r}, 5FU^{s}; marker exchange deletion	This study
JW9517	JW9516, ΔDVU2210 ΔDVU1975 Δ(P_{npt}-*npt-upp*); Km^{s}; 5FU^{r}; markerless deletion	This study
JW9518	JW9479, ΔDVU2210 ΔDVU2305-6::(P_{npt}-*npt-upp*); Km^{r}, 5FU^{s}; marker exchange deletion	This study
JW9519	JW9518, ΔDVU2210 ΔDVU2305-6 Δ(P_{npt}-*npt-upp*); Km^{s}; 5FU^{r}; markerless deletion	This study
JW9520	JW9253, ΔDVU2305-6 ΔDVU1975::(P_{npt}-*npt-upp*); Km^{r}, 5FU^{s}; marker exchange deletion	This study
JW9521	JW9520, ΔDVU2305-6 ΔDVU1975 Δ(P_{npt}-*npt-upp*); Km^{s}; 5FU^{r}; markerless deletion	This study
JW9522	JW9507, Δ*sat* ΔDVU1975 ΔDVU2305-6::(P_{npt}-*npt-upp*); Km^{r}, 5FU^{s}; marker exchange deletion	This study
JW9523	JW9522, Δsat ΔDVU1975 ΔDVU2305-6 Δ(P_{npt}-*npt-upp*); Km^{s}; 5FU^{r}; markerless deletion	This study
JW9524	JW9517, ΔDVU2210 ΔDVU1975 ΔDVU2305-6::(P_{npt}-*npt-upp*); Km^{r}, 5FU^{s}; marker exchange deletion	This study
JW9525	JW9524, ΔDVU2210 ΔDVU1975 ΔDVU2305-6 Δ(P_{npt}-*npt-upp*); Km^{s}; 5FU^{r}; markerless deletion	This study
JW9526	JW9511, Δ*sat* ΔDVU2210 ΔDVU1975 ΔDVU2305-6::(P_{npt}-*npt-upp*); Km^{r}, 5FU^{s}; marker exchange deletion	This study
JW9527	JW9526, Δ*sat* ΔDVU2210 ΔDVU1975 ΔDVU2305-6 Δ(P_{npt}-*npt-upp*); Km^{s}; 5FU^{r}; markerless deletion	This study

**Plasmids**		
pCR8/GW/TOPO	Cloning vector containing *aadAI* and pUC *ori* cassette; Sp^r^, non-replicating in *D. vulgaris* Hildenborough	Invitrogen
pCR4-TOPO	Cloning vector containing *npt*; Km^r^	Invitrogen
pMO746	pCR4-TOPO containing *upp* in an artificial operon with *npt* under the *npt* promotor; Km^r^	(Parks et al., 2013)
pMO9250	pCR8/GW/TOPO *aadAI* and pUC *ori* cassette plus 660bp region upstream of DVU2305 followed by the synthetic operon P_{npt}-*npt-upp* from pMO746 and a 633bp fragment downstream of DVU2306; Km^r^; for marker-exchange deletion of DVU2305-6, Δ(DVU2305-6)::(P_{npt}-*npt-upp*)	This study
pMO9252	pCR8/GW/TOPO *aadAI* and pUC *ori* cassette plus 660bp region upstream of DVU2305 followed by a 633bp fragment downstream of DVU2306; Sp^r^; for markerless deletion of DVU2305-6	This study
pMO9476	pCR8/GW/TOPO *aadAI* and pUC *ori* cassette plus 493bp region upstream and 4 bp of DVU2210 followed by the synthetic operon P_{npt}-*npt-upp* from pMO746 and 501bp region downstream of DVU2210; Km^r^; for marker-exchange deletion of DVU2210 ΔDVU2210::(P_{npt}-*npt-upp*)	This study
pMO9478	pCR8/GW/TOPO *aadAI* and pUC *ori* cassette plus 493bp region upstream and 4bp of DVU2210 followed by a 501bp fragment downstream of DVU2210; Sp^r^; for markerless deletion of DVU2210	This study
pMO9482	pCR8/GW/TOPO *aadAI* and pUC *ori* cassette plus 493bp region upstream of DVU2210 followed by DVU2210 and 501bp downstream; Sp^r^; for complementation of the deleted DVU2210	This study
pMO9483	pCR8/GW/TOPO *aadAI* and pUC *ori* cassette plus 493bp region upstream of DVU2210 followed by DVU2210(A583C) and 501bp downstream; Sp^r^; for site-directed mutation construct of DVU2210.	This study
pMO9502	pCR8/GW/TOPO *aadAI* and pUC *ori* cassette plus 500bp region 67bp upstream of DVU1975 followed by the synthetic operon P_{npt}-*npt-upp* from pMO746 and 453bp region 48bp downstream of DVU1975; Km^r^; for marker-exchange deletion of DVU1975 ΔDVU1975::(P_{npt}-*npt-upp*)	This study

pMO9504	pCR8/GW/TOPO *aadAI* and pUC *ori* cassette plus 500bp region 67bp upstream of DVU1975 followed by a 453bp region 48bp downstream of DVU1975; Sp^r; for markerless deletion of DVU1975	This study

[a]Definitions: WT: wild-type; *upp*: uracil phosphoribosyltransferase (gene locus ID DVU1025); *sat*: sulfate adenylyl transferase (gene locus ID DVU1295); *npt*: neomycin phosphotransferase II, confers resistance to kanamycin, contains a mutation of C$\rightarrow$A at -34bp; *aad*A1: aminoglycoside adenyltransferase with its native promotor, confers resistance to spectinomycin. $5FU^r$: 5-fluorouracil resistance due to absence of *upp* gene; Km^r: kanamycin resistance, Sp^r: spectinomycin resistance, $DVU2210_{A583C}$: variant of DVU2210 in which the adenine at nucleotide 583 is changed to cytosine causing an amino acid change of S195R in the encoded protein.

Supplementary Table 2. Primer Information

**Primer name**	**Primer sequence (5′-3′)[a]**	**Purpose**
SpecRpUC-F	CCAGCCAGGACAGAAATGCCTCG	Amplification of plasmid backbone (*aadAI*-pUC) from pCR8/GW/TOPO
SpecRpUC-R	ATGTGAGCAAAAGGCCAGCAAAAGGC	Amplification of plasmid backbone (*aadAI*-pUC) from pCR8/GW/TOPO
Kan gene Prom Nterm	CCGGAATTGCCAGCTGGGGCGC	Amplification of (P_{npt}-*npt-upp*) from pMO746
upp gene Cterm	CTTACTTGGTGCCGAATATCTTGTCGC	Amplification of (P_{npt}-*npt-upp*) from pMO746
SpecRpUC-up	CGCCTGGTATCTTTATAGTCCT	Sequencing of cloned regions
pMO719-XbaI-dn	TGGGTTCGTGCCTTCATCCG	Sequencing of cloned regions
DVU1975-upF	GCCTTTTGCTGGCCTTTTGCTCACAT<br>GTTCTCGACGACATCAAGCCCG	Amplification of upstream region of DVU1975
DVU1975-upR	GCGACAAGATATTCGGCACCAAGTAAG<br>TAGTTTTGTTCCTCGACTCGAACTTGCATC	Amplification of upstream region of DVU1975, specific for marker-exchange deletion
DVU1975-dnF	GCGCCCCAGCTGGCAATTCCGG<br>GGCACTTTCGTGTCTTGTAACGCC	Amplification of downstream region of DVU1975
DVU1975-dnR	CGAGGCATTTCTGTCCTGGCTGG<br>AACGGCGAAAGGTACGAGGC	Amplification of downstream region of DVU1975, specific for marker-exchange deletion
DVU1975-MLD-upR	GGCGTTACAAGACACGAAAGTGCC<br>TAGTTTTGTTCCTCGACTCGAACTTGCATC	Amplification of upstream region of DVU1975,

		specific for marker-less deletion
DVU1975-MLD-dnF	GATGCAAGTTCGAGTCGAGGAACAAAACTA<br>GGCACTTTCGTGTCTTGTAACGCC	Amplification of downstream region of DVU1975, specific for marker-less deletion
DVU2210-upF	GCCTTTTGCTGGCCTTTTGCTCACAT<br>GCTTCCTGCGCAAGGCGT	Amplification of upstream region of DVU2210 for deletion plasmids and upstream-DVU2210-downstream for complementation and SNP strains
DVU2210-upR	GCGACAAGATATTCGGCACCAAGTAAG<br>TCATGGGTGTTTGTGGTGCAGAC	Amplification of upstream region of DVU2210, specific for marker-exchange deletion
DVU2210-dnF	GCGCCCCAGCTGGCAATTCCGG<br>CCCTGTACCTCGGCGTGTAG	Amplification of downstream region of DVU2210, specific for marker-exchange deletion
DVU2210-dnR	CGAGGCATTTCTGTCCTGGCTGG<br>CGAGACGGAGATGGACCGC	Amplification of downstream region of DVU2210 for deletion plasmids and upstream-DVU2210-downstream for complementation and SNP strains
DVU2210-MLD-upR	CTACACGCCGAGGTACAGGG<br>TCATGGGTGTTTGTGGTGCAGAC	Amplification of upstream region of DVU2210, specific for marker-less deletion
DVU2210-MLD-dnF	GTCTGCACCACAAACACCCATGA<br>CCCTGTACCTCGGCGTGTAG	Amplification of downstream region of DVU2210, specific for marker-less deletion
DVU2210-S195R-F	CGAAGAGGAGTCCATCCTTCAAGGC***C***<br>GTTGCGAACTTGTGGAACGCCA	Introduction of A583C in DVU2210 conferring S195R; for introducing the DVU2210 variant into the marker-exchange mutant
DVU2210-S195R-R	TGGCGTTCCACAAGTTCGCAAC***G***<br>GCCTTGAAGGATGGACTCCTCTTCG	Introduction of A583C in DVU2210 conferring S195R; for introducing the DVU2210 variant into the marker-exchange mutant

DVU2305-6-upF	GCCTTTTGCTGGCCTTTTGCTCACAT<br>CCGTCAGACCCGGCTGAAG	Amplification of upstream region of DVU2305-6
DVU2305-6-upR	GCGACAAGATATTCGGCACCAAGTAAG<br>GCCTCTCTCCTGCTGTGGC	Amplification of upstream region of DVU2305-6, specific for marker-exchange deletion
DVU2305-6-dnF	GCGCCCCAGCTGGCAATTCCGG<br>AGGCAGCAGACACGACAATATCAGAAG	Amplification of downstream region of DVU2305-6
DVU2305-6-dnR	CGAGGCATTTCTGTCCTGGCTGG<br>CACGAGTGCTACATCGATATCCAGTACGT	Amplification of downstream region of DVU2305-6, specific for marker-exchange deletion
DVU2305-6-MLD-upR	CTTCTGATATTGTCGTGTCTGCTGCCT<br>GCCTCTCTCCTGCTGTGGC	Amplification of upstream region of DVU2305-6, specific for marker-less deletion
DVU2305-6-MLD-dnF	GCCACAGCAGGAGAGAGGC<br>AGGCAGCAGACACGACAATATCAGAAG	Amplification of downstream region of DVU2305-6, specific for marker-less deletion

[a]Underlined region of primer is overhang used for SLIC with plasmid backbone or P_{npt}-*npt-upp*; Underlined, bolded, and italicized ***letter*** indicates nucleotide changed in DVU2210 variant.

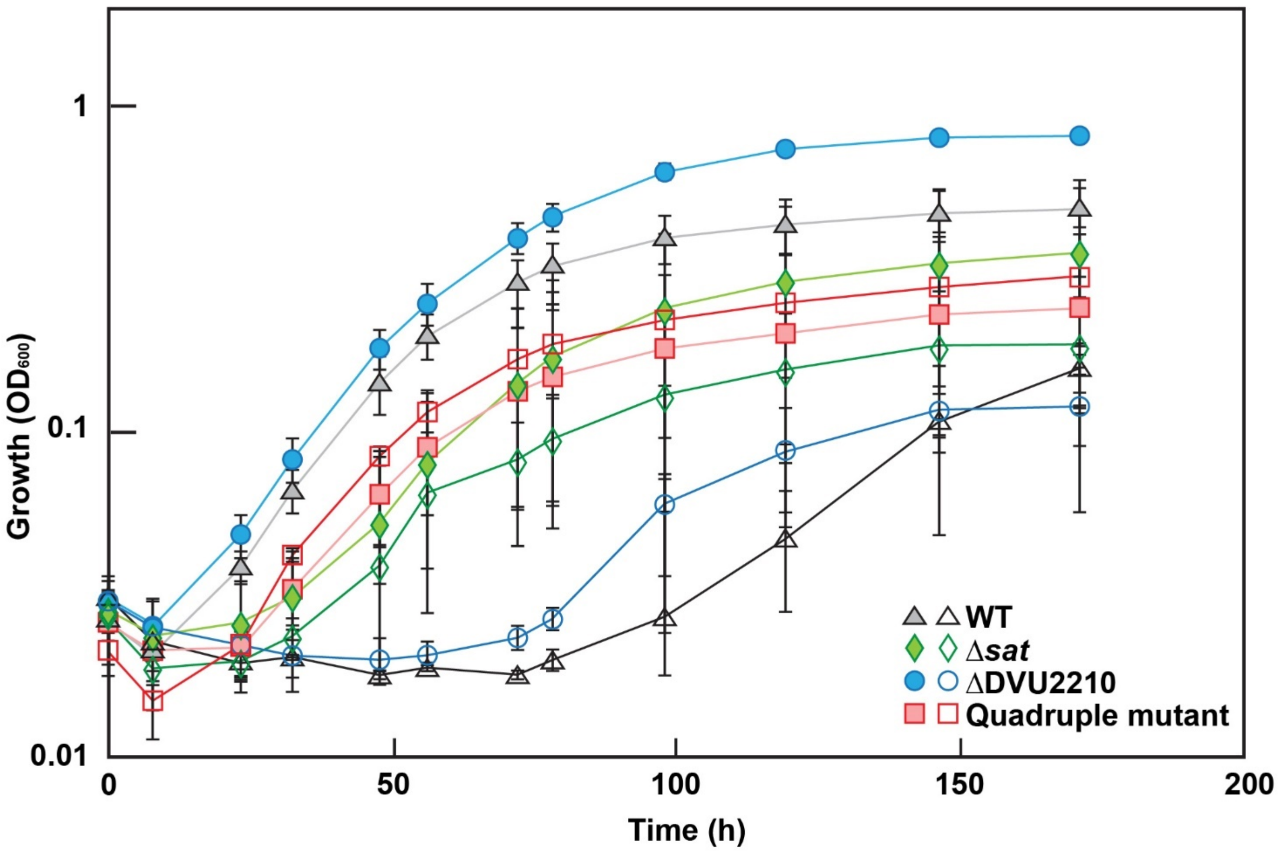

Supplementary Figure 1. Growth of *Desulfovibrio vulgaris* strains with sodium tungstate. Wild-type (WT) and mutant *D. vulgaris* strains were grown fermentatively at 34ºC on MOYPc with (open symbols) and without (closed symbols) an added 5 mM tungstate. The quadruple mutant was lacking *sat*, DVU2210, DVU1975, and DVU2305-6. Growth was measured by optical density at 600nm (OD_{600}). Error bars denote the standard deviation from the average of triplicate cultures.

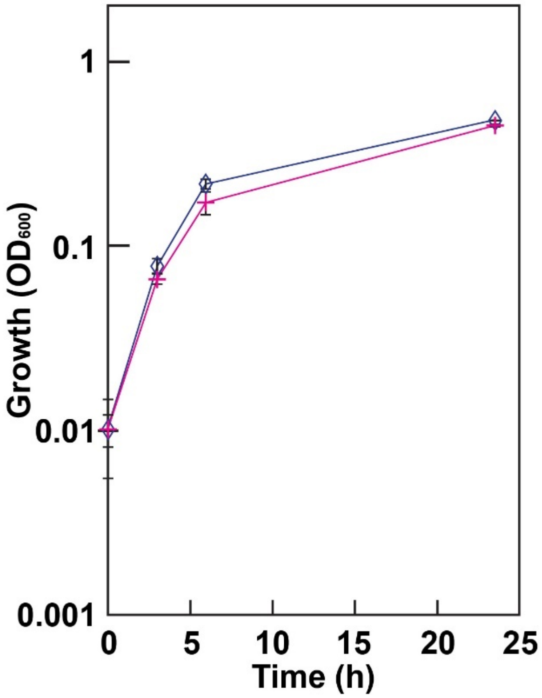

Supplementary Figure 2. Growth of *Clostridium sporogenes* ATCC 7955 with and without molybdate. *C. sporogenes* was grown anoxically in a modified Tanner's medium with no additional molybdate (diamonds) or an additional 7.5 mM sodium molybdate (crosses). Medium (10 ml) was inoculated with 0.2 ml of an overnight culture and incubated statically at 30ºC. The modified Tanner's medium (Tanner, 2007) contained the following per liter: 0.8 g sodium chloride, 1 g ammonium chloride, 0.1 g potassium chloride, 0.1 g potassium phosphate monobasic, 0.2 g magnesium sulfate heptahydrate, 0.04 g calcium chloride dihydrate, 5 g glucose, 2 g yeast extract, 10 g TES, 1 ml vitamins, 5 ml trace elements. The vitamin solution was made at a 10x concentration of that described in Brandis and Thauer (1981). The trace elements solution was the same as that described for the MO medium in this study. All solutions were made in anoxic milliQ water generated by boiling the water under nitrogen gas. Growth was measured by optical density at 600nm (OD_{600}). Error bars denote the standard deviation from the average of triplicate cultures.

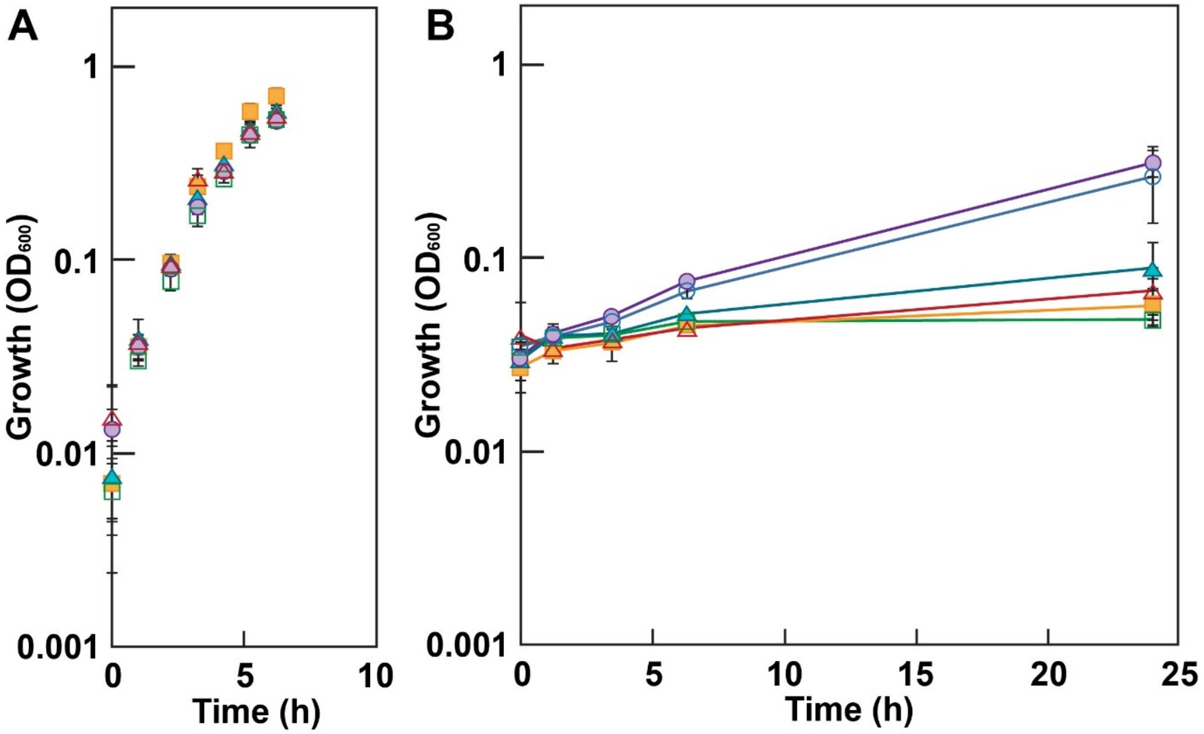

Supplementary Figure 3. *E. coli* growth in the presence of sodium molybdate. *E. coli* cultures were grown on LC (A) or M9 medium (Atlas, 2010) with 1.25 µM $FeCl_2$ (B) with 0, 3, or 10 mM sodium molybdate (designated by circles, triangles, and squares, respectively). Two *E. coli* strains were analyzed, one containing the *D. vulgaris* wild-type DVU2210 on plasmid pMO9582 (open symbols) and the other containing the modified DVU2210 (A583C) on plasmid pMO9583 (closed symbols). Starter cultures were prepared by inoculating a scrape from a frozen stock into 50 ml flasks containing 5 ml of LC with spectinomycin. These were incubated at 37ºC and shaken at 150 rpm. Following overnight incubation, 1 ml of each culture was washed to remove medium components by centrifuging the cells at 17,000 x g, removing the supernatant, resuspending the pellet in 1 ml of phosphate buffered saline pH 7.4, and repeating centrifugation and resuspension. Test tubes containing 3 ml of medium were inoculated with 0.03 ml of the washed starter cultures and incubated at 37ºC while shaking at 150 rpm. Growth was measured by optical density at 600nm (OD_{600}). Error bars denote the standard deviation from the average of triplicate cultures.

REFERENCES

Atlas, R. M. (2010). *Handbook of Microbiological Media*. 4th ed. Boca Raton, FL: CRC Press.

Brandis, A., and Thauer, R. K. (1981). Growth of *Desulfovibrio* species on hydrogen and sulphate as sole energy source. *J. Gen. Microbiol.* 126, 249–252.

Hillesland, K. L., Lim, S., Flowers, J. J., Turkarslan, S., Pinel, N., Zane, G. M., et al. (2014). Erosion of functional independence early in the evolution of a microbial mutualism. *Proc. Natl. Acad. Sci. USA* 111, 14822–14827. doi:10.1073/pnas.1407986111.

Keller, K. L., Bender, K. S., and Wall, J. D. (2009). Development of a markerless genetic exchange system for *Desulfovibrio vulgaris* Hildenborough and its use in generating a strain with

increased transformation efficiency. *Appl. Environ. Microbiol.* 75, 7682–7691. doi:10.1128/AEM.01839-09.

Parks, J. M., Johs, A., Podar, M., Bridou, R., Hurt, R. A., Jr., Smith, S. D., et al. (2013). The genetic basis for bacterial mercury methylation. *Science* 339, 1332–1335. doi:10.1126/science.1230667.

Tanner, R. S. (2007). "Cultivation of Bacteria and Fungi," in *Manual of Environmental Microbiology*, eds. C. J. Hurst, R. L. Crawford, J. L. Garland, D. A. Lipson, A. L. Mills, and L. D. Stetzenbach (Washington, D.C.: ASM Press), 69–78.
